# Assessing Different Mechanisms of Toxicity in Mountaintop Removal/Valley Fill Coal Mining-Affected Watershed Samples Using *Caenorhabditis elegans*

**DOI:** 10.1371/journal.pone.0075329

**Published:** 2013-09-16

**Authors:** Elena A. Turner, Gretchen L. Kroeger, Mariah C. Arnold, B. Lila Thornton, Richard T. Di Giulio, Joel N. Meyer

**Affiliations:** Nicholas School of the Environment, Duke University, Durham, North Carolina, United States of America; Warren Alpert Medical School of Brown University, United States of America

## Abstract

Mountaintop removal-valley fill coal mining has been associated with a variety of impacts on ecosystem and human health, in particular reductions in the biodiversity of receiving streams. However, effluents emerging from valley fills contain a complex mixture of chemicals including metals, metalloids, and salts, and it is not clear which of these are the most important drivers of toxicity. We found that streamwater and sediment samples collected from mine-impacted streams of the Upper Mud River in West Virginia inhibited the growth of the nematode *Caenorhabditis elegans*. Next, we took advantage of genetic and transgenic tools available in this model organism to test the hypotheses that the toxicity could be attributed to metals, selenium, oxidative stress, or osmotic stress. Our results indicate that in general, the toxicity of streamwater to *C. elegans* was attributable to osmotic stress, while the toxicity of sediments resulted mostly from metals or metalloids.

## Introduction

Mountaintop removal/valley fill coal mining is a form of surface mining wherein all vegetation and topsoil are removed, overlying rock is dynamited to expose coal seams, and spoilage is moved into adjacent valleys, thus burying streams [1]. While mountaintop removal/valley fill coal mining (herein abbreviated as MTR/VF) offers a lower-cost way to extract coal than traditional shaft mines, full risk-benefit assessments of this practice require an understanding of potential ecological and human health effects [2]. However, despite the fact that a thorough understanding of those health effects is currently lacking, MTR/VF is already occurring on a very large scale. Lindberg et al. recently estimated the total extent of surface mining as 6.8% of Appalachian coal fields, or 4.68 million square hectares [3].

Evaluating the potential toxic effects of MTR/VF is complicated by the facts that the effluent from such sites is highly complex and variable in space and time. The “TRIAD” approach, which utilizes a combination of chemistry, bioassays and *in situ* studies, has been proposed to assess the potential health effects of such complex environmental pollutant mixtures. This approach takes advantage of the complementary strengths of field surveys, analytical chemistry, and in-laboratory analysis of the toxicity of field-collected samples [4]. Field surveys of the environmental effects of MTR/VF on stream ecology and benthic invertebrate communities are emerging [5-7], and surface mining has also been linked to negative impacts on human health [8,9]. Analytical chemistry approaches have documented high levels of inorganic solutes in waters and sediments downstream from MTR/VF sites [3,5,10,11]. However, the body of laboratory-based research investigating the toxicity of mine-impacted water and stream sediment is smaller [3,11,12], and currently lacks a strong mechanistic component.

To strengthen our understanding of the potential toxicity of MTR/VF-associated water and sediment samples, we utilized *Caenorhabditis elegans*. This genetically well-characterized model organism is being increasingly used in environmental toxicology research to gain mechanistic insight into environmental samples which may operate via one or several different mechanisms of toxicity [13-15]. *C. elegans* normally inhabits moist, microbe-rich soil surface environments [16], but is routinely cultured for multiple generations in liquid medium [17]and frequently used for aquatic toxicity tests [12,14,18,19]. Furthermore, while there are many other invertebrate species available for aquatic toxicology testing, none offer the same genetic tools. *C. elegans* shares stress-response pathways with higher eukaryotes [20,21], and many mutant and reporter strains that facilitate mechanistic analysis are available and widely used. For example, if knockout mutants that do not produce crucial metal-chelating peptides are hyper-sensitive to exposure to a particular environmental mixture, this suggests that this mixture is causing toxicity in part due to metal content. The same approach can be used to diagnose exposures that cause toxicity via DNA damage, oxidative stress, and other mechanisms using the appropriate mutant strains [19,22-24], an approach that has been dubbed “functional toxicology” [25]. Similarly, transgenic strains that carry a “reporter” such as green fluorescent protein (GFP) driven by the promoter region of stress-response genes can provide a stress-response endpoint at lower thresholds than those that cause outright toxic effects [12,26].

We used *C. elegans* to investigate the toxicity and mechanism of toxicity of environmental samples from the Mud River and several associated tributaries (Boone County, Southwestern West Virginia). The Mud River flows through the Hobet 21 surface mine from which it receives numerous discharges, both directly and via its tributaries; the sampling sites have previously been characterized with respect to water chemistry and show both conductivity in excess of the proposed EPA benchmark of 300µs/cm^-1^ and selenium levels in excess of EPA water quality criteria of 5µG Se L^-1^ [3]. Previous work has suggested that increased levels of metals and total dissolved solids (TDS) may be important stressors in MTR/VF systems [6]. We hypothesized that water and sediment samples from putatively mining-impacted streams would affect the growth of wild-type nematodes, and that mutant nematodes would be differentially affected based on the composition of the stream water and sediment pore water. Furthermore, we analyzed mutant strains that would help us distinguish the contributions of metals or metalloids, osmotic stress and oxidative stress to the toxicity of these samples (Table 1).

**Table 1:** Strains.

**Strain**								**Gene Knockout**												**Mechanism**			
*gpdh-1(ok1558*); *gpdh-2(kb33*)								glycerol-3 phosphate dehydrogenase												sensitive to osmotic stress			
*mtl-2(gk125*)								metallothionein												sensitive to heavy metals			
mtl-2::GFP reporter								none												produces GFP-tagged metallothionein			
N2								none												wild-type			
*pcs-1(tm1748*)								phytochelatin synthase												sensitive to metals and metalloids			
*smf-2(gk133*)								divalent metal transporter												sensitive to manganese exposure			
*sod-3(gk257*)								superoxide dismutase												sensitive to oxidative stress			

Stream water and sediment pore water from putatively mine-impacted sites along the Mud River and its tributaries had significant impacts on the growth of wild-type and mutant nematodes. Effects on growth ranged from positive to highly negative and varied between site, strain and season; additionally, stream water and sediment pore water from the same site often produced different effects on growth.

## Materials and Methods

### Sample Collection and Handling

**Figure 1:**
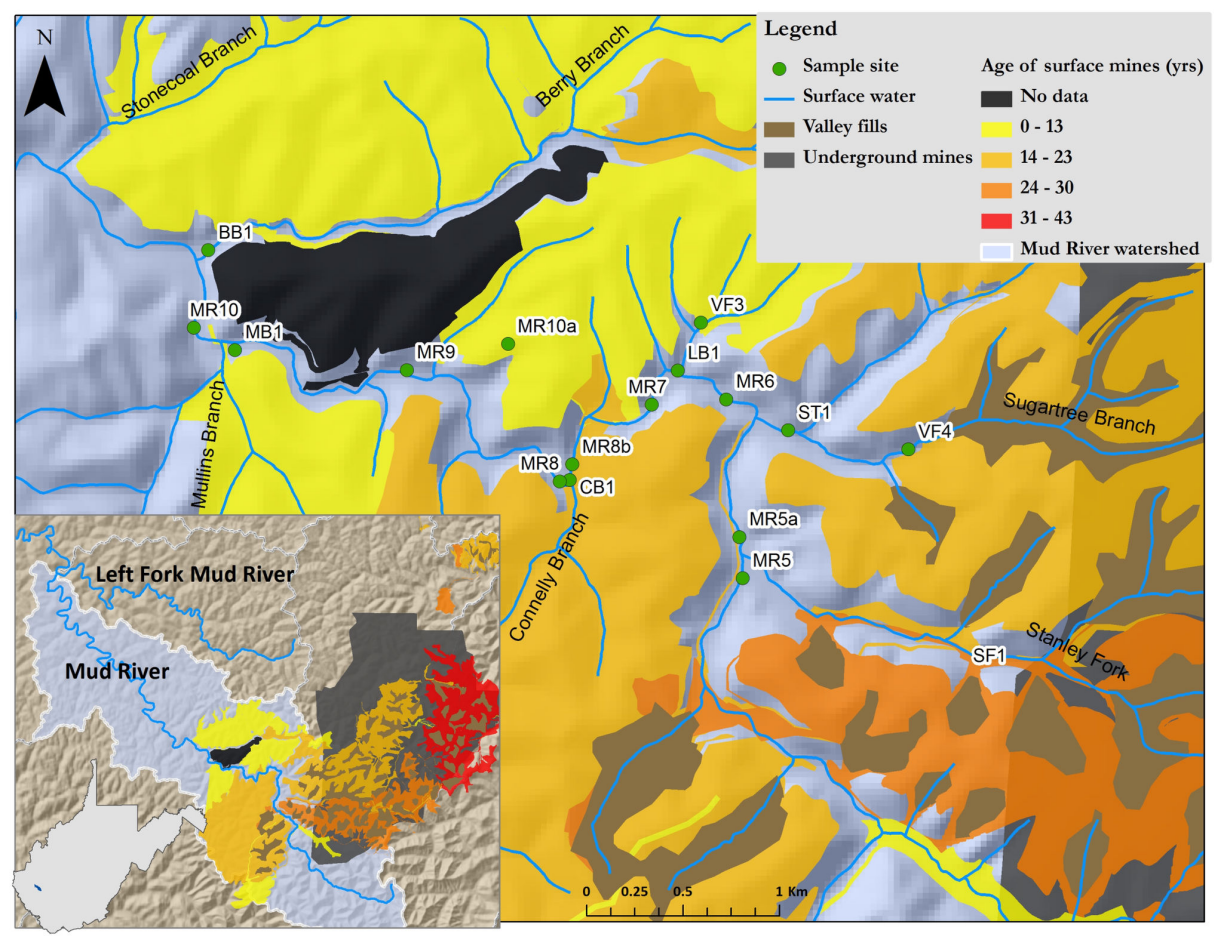
Map of the Mud River watershed. Sampling sites plus age and extent of surface mining activity. Insets show sequentially larger geographic regions.

Sample collection took place June 24 2010, December 8-10 2010, April 12-15 2011, and May 1 2012 at sites shown in Figure 1. Samples from Mud River 7 were taken on private property with the owner’s permission. All other samples were taken from easements of publicly accessible roads in accordance with local regulations. Rivers are designated as public property in West Virginia. Our field sampling did not involve endangered or protected species. Stream water samples were collected in metal-free 50 ml polypropylene tubes; sediment was collected in glass mason jars using a small plastic trowel. Stream water conductivity was measured during sediment and water collection. Water and sediment samples were stored on ice while in the field and transferred to a 4 °C cold room. Sediment was passed through a 2mm sieve and frozen in 5 to 7 ml aliquots at -80 °C until use. Pore water was prepared by thawing sediment, adding an equal volume of ddH _2_0, shaking vigorously for two minutes, and centrifuging at 6987xg for 10 minutes. The supernatant (“pore water”) was removed and used for growth assays; all pore water was stored at 4 °C until use and replicates were run within 4 to 6 weeks.

### Nematode Culture

Nematodes were maintained at 20 or 15 °C on K agar plates with a lawn of OP 50 strain *Escherichia coli* [27]. Synchronized L1 larvae were obtained by dissolving gravid adults with a solution of 5% sodium hypochlorite and hatching eggs in *K* *medium* plus MgSO_4_, CaSO_4_ and cholesterol. The *pcs-1* (*tm1748* allele, outcrossed 6x) mutant strain was obtained from Stephen Sturzenbaum (King’s College, London). The *gpdh-1* and *gpdh-2* (*ok1558* and *kb33* alleles, outcrossed 3 times) mutant strains were obtained from Todd Lamitina (University of Pennsylvania). *gpdh-1*(*ok1558*) males were generated by heat-shock and crossed with *gpdh-2*(*kb33*) hermaphrodites to generate *gpdh-1*(*ok1558*);*gpdh-2*(*kb33*) double mutants; homozygosity for both deletions was confirmed by PCR using primers designed using Wormbase (*Gpdh-1* forward primer: 5’ ccctcttcaggattcttccc 3’, reverse primer 5’ tccgctagatccatttccag 3’; *Gpdh-2* forward primer 5’ cgcgaacaaagaatatttaggaa 3’, reverse primer 5’ aattcgtcaaaggaatctgtgaa 3’). The *mtl-2* mutant (JF23 strain, *gk125* allele, outcrossed 4 times) was obtained from Jonathan Freedman (NIEHS). N2 (Bristol) wild-type and *smf-2* (VC171 strain, *gk133* allele) and *sod-3* (VC433 strain, *gk235* allele) mutant strains were obtained from the *Caenorhabditis* Genetics Center (CGC, Minneapolis, MN, USA). The *sod-3* strain was outcrossed 3 times with N2 (Bristol wildtype) males; genotyping was performed using primers designed using Wormbase (*sod-3* forward primer: 5’ gtcccgaaatgcattttttc 3’, reverse primer 5’ gggcggacatttttgtactg 3’). The *mtl-2::GFP* reporter gene strain was obtained from Phillip Williams (University of Georgia).

### Growth assays

On day 0 of each growth assay, synchronized L1 larvae were distributed onto 96-well plates (n=50) with a COPAS Biosort (Union Biometrica, Holliston MA) using 50 µL 2x EPA reconstituted moderately hard water [22]. An additional 50 µL of dosing solution composed of stream water, sediment pore water, or sodium selenite at 2x final dosing strength plus cholesterol (final concentration of 0.01 mg/mL) was added to the wells, and plates were incubated at 20 degrees for the course of the assay. Dosing solution was renewed daily. Worms were fed UVC-killed *E. coli* (UVRA strain) to eliminate the potentially confounding effect of bacterial metabolism on exposures, as previously described [19]. Bacteria were concentrated to approximately 1x10^10^ cells/mL [28], at 1:100 by volume for the first day, 1:50 for the second day and 1:10 for the final day of the growth assay. Increasing concentrations of bacteria were added to accommodate the increasing food requirements of the growing nematodes. Growth of the worms was measured using a COPAS Biosort, using extinction (optical density of the worm) as a growth endpoint [22]. Extinction data from the third day of exposure was graphed as a percentage of the growth of EPA water exposed controls. For the purposes of this paper we define 3-day exposures as medium-term, because they encompass most of the developmental trajectory of this species.

### Reporter strain assay

50 L4/young adult (staged as having minimally reached the L4 stage based on appearance of the vulval crescent, but not having reached reproductive adulthood based on absence of eggs before and after exposure) *mtl-2::GFP* nematodes were distributed into 24 well plates containing sediment pore water, EPA water, 0.5µM cadmium chloride, 5mM manganese chloride or 0.15µM sodium selenite for a total volume of 500µL, with no food. Plates were incubated at 20 °C for 24 hours, and fluorescence was measured with the COPAS Biosort and graphed as a ratio of GFP expression to extinction (as a proxy for size).

### Statistical analysis

Size (growth) and fluorescence measurements were analyzed via Mann Whitney U tests conducted using Statview for Windows (Version 5.0.1, SAS Institute Inc., Cary, NC). Bonferroni corrections were applied to p value cut-offs to determine significance. For example, for Figure 1a, 13 exposures were compared to the EPA water control, so that the cut-off for significance was p < 0.0038 (0.05/13) rather than p < 0.05.

## Results

### I: Mud River and tributary samples affected the growth of wild-type nematodes

**Figure 2:**
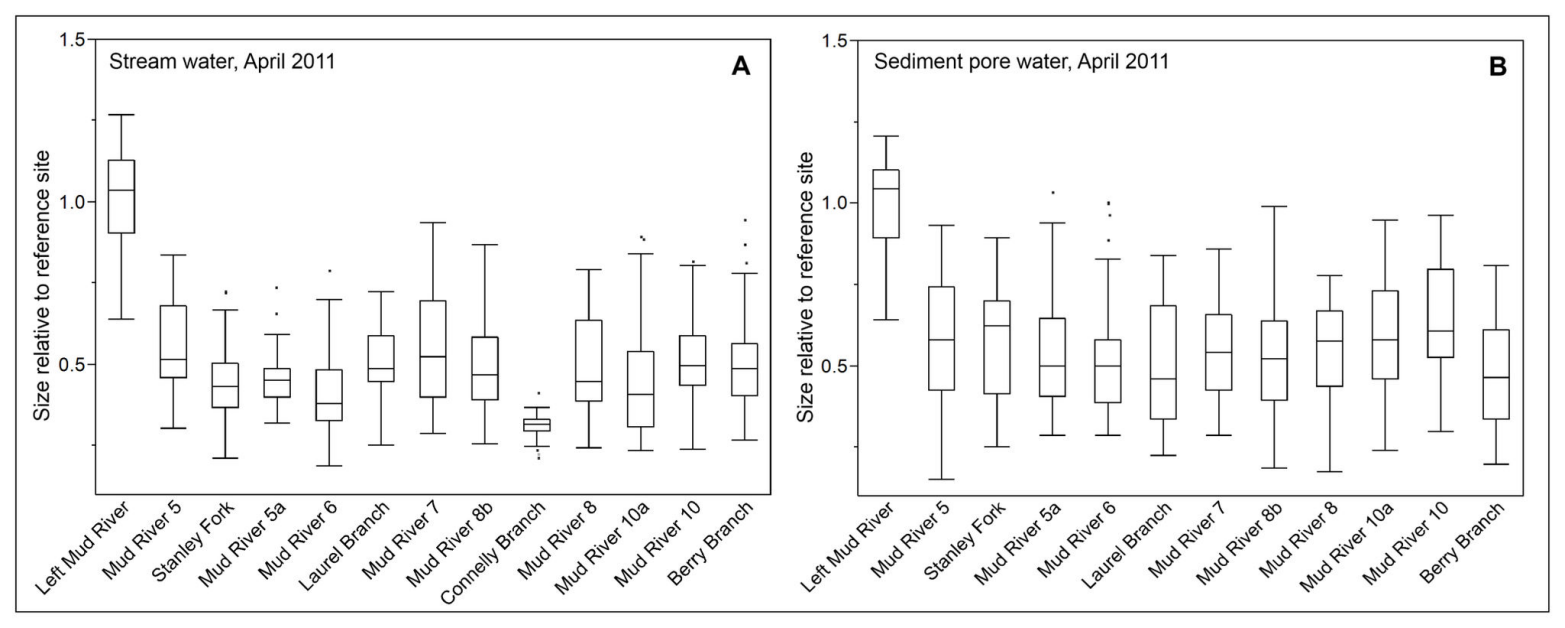
Growth effects of stream water and sediment pore water. The growth of wild-type nematodes was affected by stream water (**2A**, n=27-359) and sediment pore water (**2B**, n=30-359) from the Mud River and its tributaries, but not by water or sediment pore water from a reference site on the unmined Left Fork. Statistically significant difference from EPA water control indicated by asterisks. “Size” refers to the optical density (extinction) of each nematode and describes growth since the data shown is for size after three days of exposure. Each exposure was repeated at least two times separately. EPA water was used as a laboratory control medium.

We exposed wild-type nematodes to water and sediment pore water from one non-mining impacted site and multiple impacted sites shown in Figure 1. Both stream water and sediment pore water from mining-impacted sites on the Mud River and its tributaries reduced the growth of wild-type nematodes in comparison to EPA water controls (Figure 2). Lethality was not observed for any of the exposure conditions. Reduction in growth varied between sites but was statistically significant for all water samples from mining-impacted sites and three sediment pore water samples from mining-impacted sites sampled in April 2011 (Figure 2 and Table 2). Water from a reference site on the unmined left fork of the Mud River did not reduce the growth of wild-types nematodes; in fact, there was a slight positive effect on growth in wild-type nematodes exposed to sediment pore water from the reference site in comparison to EPA water-treated controls. Because growth of wild-type nematodes was better in the reference site water than in EPA MHRW, we carried out statistical analyses for all experiments with wild-type nematodes using the both the reference site and EPA MHRW for comparison (p values reported in Table 2 and Table S1, respectively).

**Table 2:** *p* values, Mann Whitney U test in comparison to reference site.

April 2011			
**Site**	**Water**	**Sediment Pore Water**	**Water vs. Sediment Pore Water**
**Left Mud River (reference**)	−	−	0.1775
**Mud River 5**	<0.0001	<0.0001	0.3939
**Stanley Fork**	<0.0001	<0.0001	<0.0001
**Mud River 5a**	<0.0001	<0.0001	0.0237
**Mud River 6**	<0.0001	<0.0001	<0.0001
**Laurel Branch**	<0.0001	<0.0001	0.8584
**Mud River 7**	<0.0001	<0.0001	0.7772
**Mud River 8b**	<0.0001	<0.0001	0.0741
**Connelly Branch**	<0.0001	<0.0001	----
**Mud River 8**	<0.0001	<0.0001	0.0042
**Mud River 10a**	<0.0001	<0.0001	<0.0001
**Mud River 10**	<0.0001	<0.0001	0.0001
**Berry Branch**	<0.0001	<0.0001	0.7691
p-value cutoff for statistical significance	0.0042	0.004545	0.0042

Comparison of optical density (extinction) of wild-type nematodes treated with steam water and sediment pore water to EPA water-treated controls. The Bonferroni-corrected p-value cut-off for significance based on 12 pairwise comparisons is p < o.oo42.

The fact that *C. elegans* grew less well in EPA MHRW than in water from our reference site is not surprising. Other researcher have found that the common aquatic toxicology species *Hyallela* *azteca* , for example, grows relatively poorly in this medium, perhaps due to insufficient chloride [29,30], although bromide [31] or other elements may also be important. Since selenium is a known contaminant of the Mud River [3] and a micronutrient [32], we next investigated its effects on the growth of wild-type nematodes.

### II: Selenium stimulates the growth of *C. elegans* at low doses, but is toxic at higher doses

**Figure 3:**
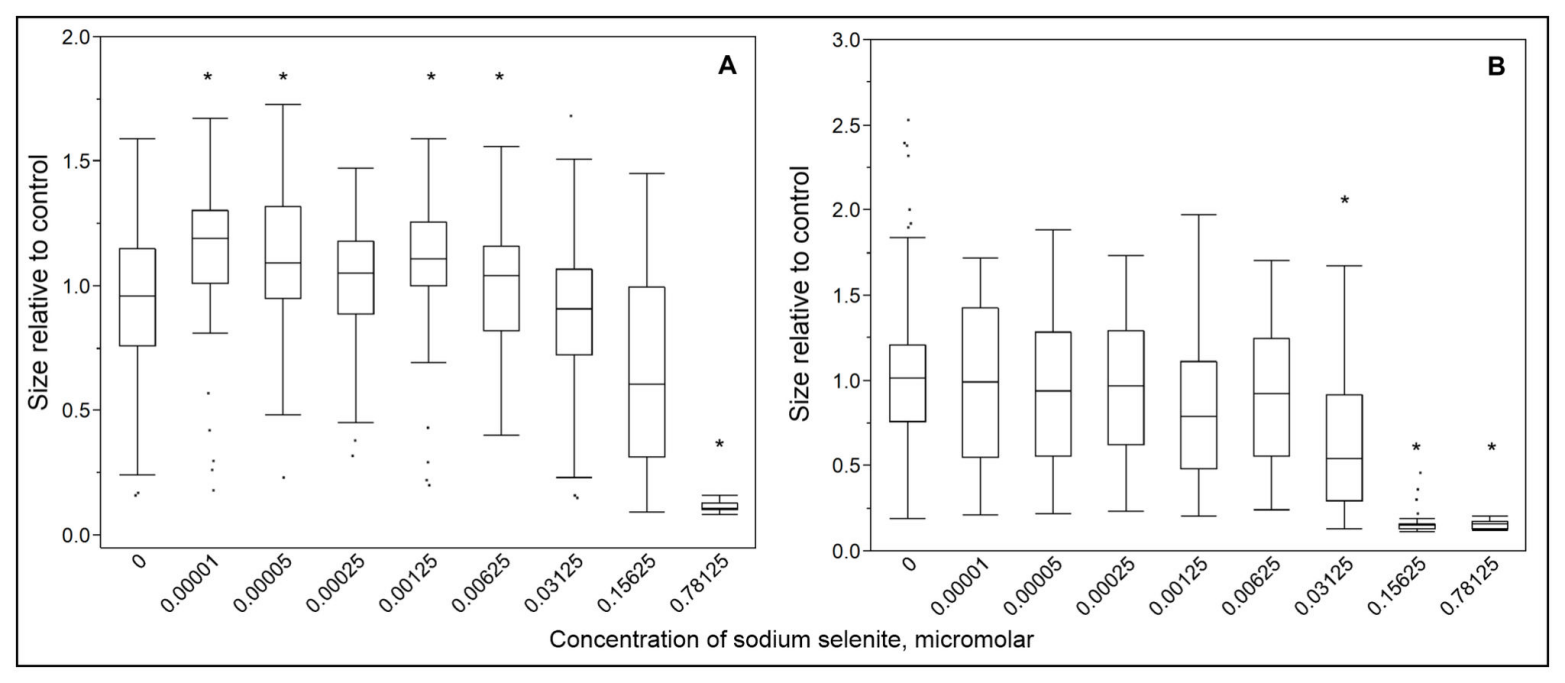
Growth of wild-type and *pcs-1* knockout nematodes was affected by sodium selenite. The growth of wild-type (**3A**, n=49-247) and *pcs-1*(tm1748) knockout (**3B**, n=30-279) nematodes was impacted both positively and negatively by addition of sodium selenite to EPA water, according to a non-monotonic dose-response curve. Statistically significant difference from EPA water control indicated by asterisks. Negative effects on growth were seen at lower doses in *pcs-1*(tm1748) mutants than in wild-type worms, and positive effects were not observed in the *pcs-1*(tm1748) strain. “Size” refers to the optical density (extinction) of each nematode and describes growth since the data shown is for size after three days of exposure. Each exposure was repeated at least two times separately. Please note the difference in Y-axis scaling.

Though the toxic effects of the metalloid selenium in aquatic invertebrates are well-documented [6,33-35], there is considerably less published work on the toxicity of selenium to nematodes. The mechanism of toxicity of selenium in nematodes is not well studied, though oxidative stress-induced cholinergic neurodegeneration has been demonstrated [36]. While toxic at moderate to high doses, selenium is also an essential micronutrient and previous research demonstrated positive effects on the growth and development of nematodes exposed to very low concentrations of selenite [32,37]. To test whether the same phenomenon could be occurring under our experimental conditions, we performed growth assays exposing worms to a wide range of concentrations of sodium selenite. Very low concentrations (0.0001-0.00005µM) of sodium selenite caused a small but significant increase in growth in treated worms compared to untreated controls (Figure 3A). Positive effects on growth became less pronounced as concentration increased; concentrations in the 0.15625 to 0.78125µM range caused highly variable growth and concentrations above 0.5µM caused total growth arrest or death (lethal doses not shown on graph).

Since subsequent tests for toxicity were performed using the *pcs-1* mutant strain which cannot synthesize phytochelatin, and since phytochelatin has been shown to bind selenium [38], we also tested the effect of selenite exposure in the *pcs-1*(*tm1748*) deletion mutants. Positive growth effects were not statistically significant for most low concentrations in *pcs-1*(*tm1748*) deficient nematodes treated with sodium selenite. Overall variability in growth was greater at all concentrations in *pcs-1*(*tm1748*) than wild-type nematodes, and overt toxicity was observed at lower concentrations in *pcs-1*(*tm1748*) than in wild-type worms (Figure 3b). Comparable concentrations of sodium selenate caused no effect on the growth of either wild-type or *pcs-1*(*tm1748*) nematodes (data not shown), in keeping with the relative toxicity of these two Se species in most organisms [39]. We note that the nematodes were fed UVC-inactivated bacteria, so that alteration of the selenium to typically more-toxic organic forms [39] is unlikely. Pilot studies with autoclaved samples ruled out an alternate food source as the cause of positive growth effects (data not shown).

### III: Tributaries increased the toxicity of the main stem

**Figure 4:**
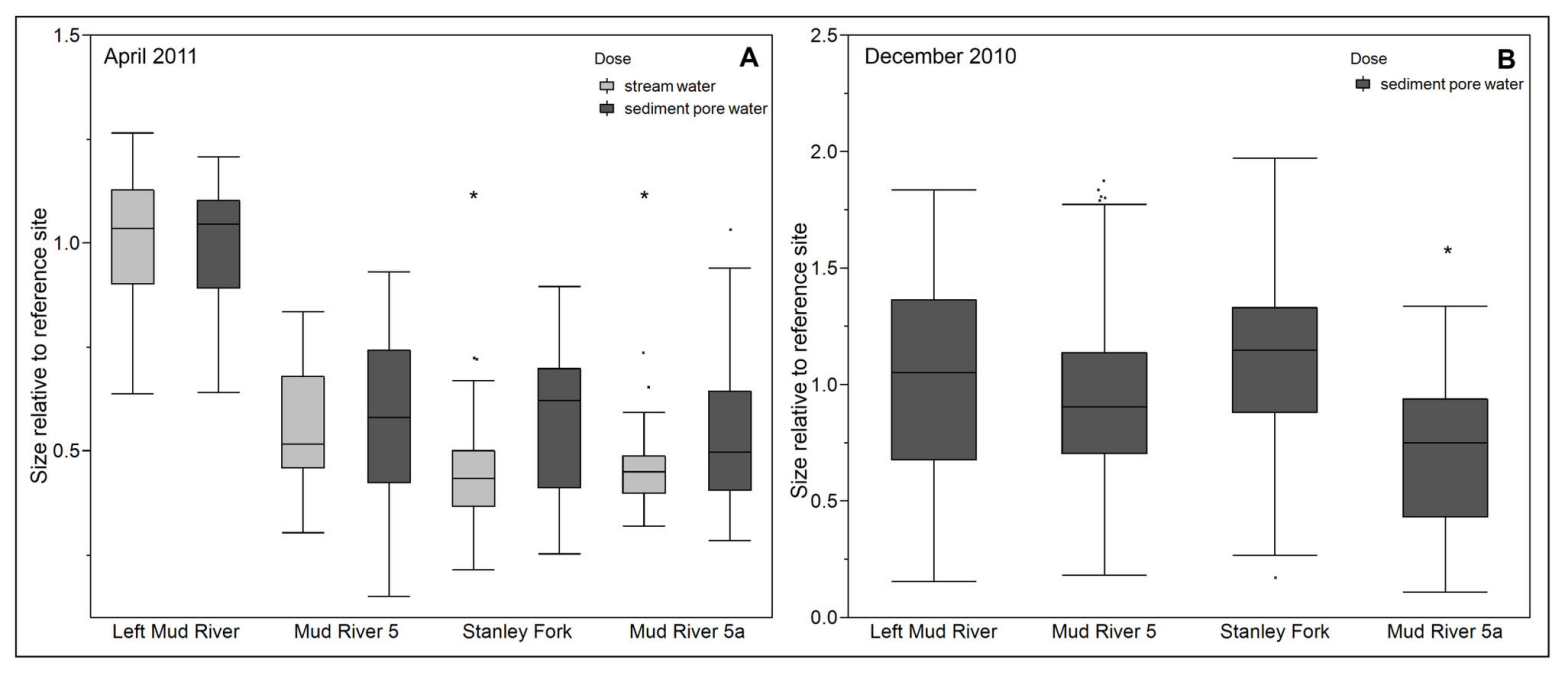
Mining-impacted tributaries affect the toxicity of the Mud River. Contributions from mine-affected tributaries have an additive effect on the toxicity of both water (**4A**, April 2011n=37-251) and sediment (**4B**, December 2010 n=148-467) from the main branch of the Mud River. Statistically significant difference between upstream and downstream site indicated by asterisks. “Size” refers to the optical density (extinction) of each nematode and describes growth since the data shown is for size after three days of exposure. Each exposure was repeated at least two times separately. Please note the difference in Y-axis scaling.

We hypothesized that the toxicity of the water and sediment pore water in Mud River was influenced not only by discharge points along the main stem but also by contributions from its mining-impacted tributaries. Comparison of growth data from two different sampling trips supported this hypothesis; stream water from April 2011 (n=27-53) and sediment pore water from December 2010 (n=98-163) from Mud River 5a, downstream from the input of the mining-impacted Stanley Fork, caused greater growth inhibition in wild-type nematodes than stream water or sediment pore water, respectively, from the upstream site of Mud River 5 (Figure 4, p=0.0013 for April 2011, p<0.0001 for December 2010. Stream water and sediment pore water collected from the same site often caused different effects on growth in wild-type nematodes (see Table 2). Potential explanations for these differences include different quantities of potential toxicants, different mechanisms of toxicity, or changing concentration of contaminants resulting from rain events or drought conditions. Having quantified growth inhibition in wild-type nematodes, we next conducted investigations into the mechanism(s) of toxicity using GFP reporter strains and knockout mutants.

### IV: MTR/VF samples did not cause induction of *mtl-2*::*GFP*

Sediment pore water from the Mud River and two tributaries, sampled in December 2010 and April 2011, did not cause an increase in metallothionein expression in *mtl-2::GFP* transgenic nematodes (Figure S1). A statistically significant increase in GFP was induced by cadmium chloride treated positive controls (p= 0.0001). While manganese and selenium are not commonly reported to be metallothionein inducers (we found one report of induction by selenite [40]:), we also tested manganese chloride and sodium selenite because they are suspected contaminants in this system. However, we observed no induction in either case (Figure S1).

### V: Genetic approaches reveal different mechanisms of toxicity for water and sediment samples

To further investigate both the potential mechanisms of toxicity responsible for both the growth inhibition seen in wild-type nematodes and the differences observed between stream water and sediment pore water exposures, we employed a number of stress-response knockout mutants which we hypothesized would be more sensitive to potential toxicants and thus display greater growth inhibition than wild type worms. We examined strains lacking genes important in protecting this organism against metal toxicity, osmotic stress, and oxidative stress, three potential mechanisms of toxicity associated with MTR/VF samples.

Metal detoxification in nematodes is mediated by a number of different metals-sequestering peptides, including two metallothioneins, *mtl-1* and *mtl-2*, the latter of which is strongly induced in the gut following exposure to cadmium and other heavy metals [41]. Although *mtl-2* deletion mutants have often been employed as metals-sensitive biosensors using a variety of different endpoints [19,42,43], growth of *mtl-2*(*gk125*) mutants was not significantly different from wild-type when exposed to water and sediment pore water from Mud River 6, 7, 9, 10 and 11 (data not shown). in keeping with the lack of metallothionein induction seen in the sediment-exposed *mtl-2::GFP* nematodes.

**Figure 5:**
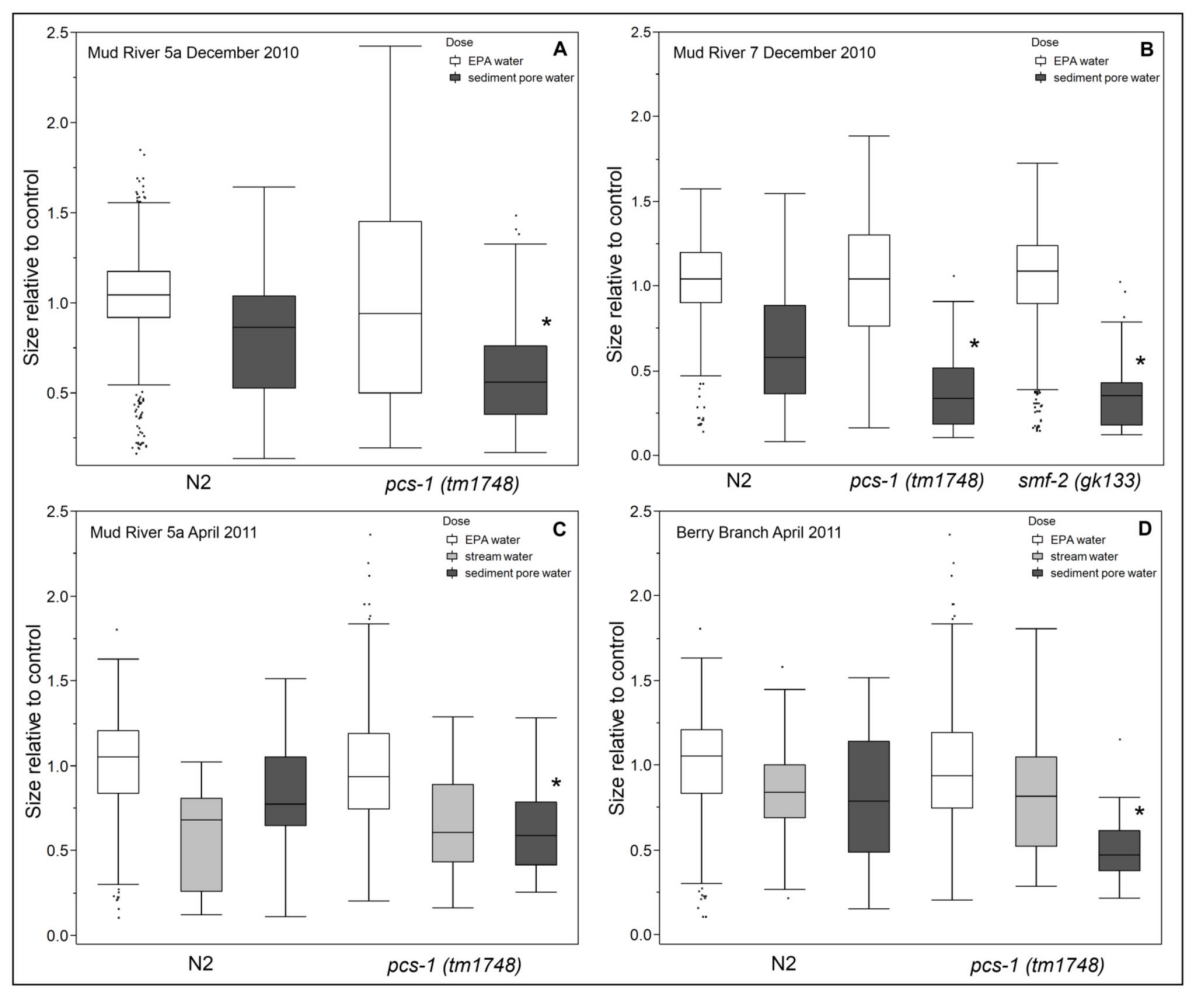
Toxicity of sediment pore water is driven by metals or metalloids. Stream sediment pore water, but not stream water, caused greater growth inhibition in metals-sensitive *pcs-1*(tm1748) and *smf-2* mutants than in wild-type nematodes, demonstrated in samples collected from Mud River sites 5a and 7 in December 2010 (**5A** p=<0.0001, n=142-533**, 5B** p=0.0022, n= 42-277), Mud River 5a in April 2011 (**5C** p=<0.0001 n=55-277) and Berry Branch in April 2011 (5D p=<0.0001, n= 125-482). Statistically significant difference from sediment-treated wild-type nematodes indicated by asterisks. “Size” refers to the optical density (extinction) of each nematode and describes growth since the data shown is for size after three days of exposure. Each exposure was repeated at least two times separately.

Phytochelatin, a cysteine-rich metal chelating peptide best studied in yeast and plants, also occurs in nematodes. Production of phytochelatin, using glutathione as a precursor, is driven by phytochelatin synthase. A deletion mutant strain lacking the phytochelatin synthase gene (*pcs-1*(*tm1748*)) is more sensitive to a number of metals than either the wild-type strain or metallothionein deletion mutants [42], and is also more sensitive to selenium (Figure 3). Growth inhibition in water-exposed *pcs-1*(*tm1748*) mutants was comparable to that of wild-type nematodes (Figure 5). However, sediment pore water from multiple river and tributary sites induced greater growth inhibition in *pcs-1*(*tm1748*) mutants than their wild-type counterparts (Figure 5, p = 0.0022 or less), indicating the presence of biologically relevant amounts of metals or metalloids in stream sediment. Lethality was not observed for any of the exposure conditions.

Manganese is another potential contaminant of interest in MTR/VF impacted rivers and streams [11,44]. Uptake and transport of manganese and iron in nematodes is regulated by the NRAMP-type divalent cation transporters *smf-1*, *smf-2* and *smf-3*. While *smf-1* and *smf-3* deletion mutants are deficient in the uptake of manganese and display greater tolerance, *smf-2*(*gk133*) deletion mutants are more sensitive to manganese exposure than wild-type worms [11,45]. Growth inhibition in *smf-2*(*gk133*) knockout mutants was similar to wild-type for most sites. *Smf-*2(*gk133*) knockout mutants were exposed to sediment pore water from Mud River 5-10, Stanley Fork, Laurel Branch and Berry Branch from December 2010, water and sediment pore water from Mud River 5-8b, 10, Stanley Fork, Laurel Branch, Berry Branch and water only from Connelly Branch from April 2011. Lethality was not observed for any of the exposure conditions. For site samples that elicited greater growth inhibition in *smf-2*(*gk133*) mutants versus wild-type worms, a similar effect was seen in *pcs-1* mutants (Figure 5b). There were no sites that produced growth-inhibited *smf-2*(*gk133*) worms without a corresponding effect in *pcs-1* mutants.

**Figure 6:**
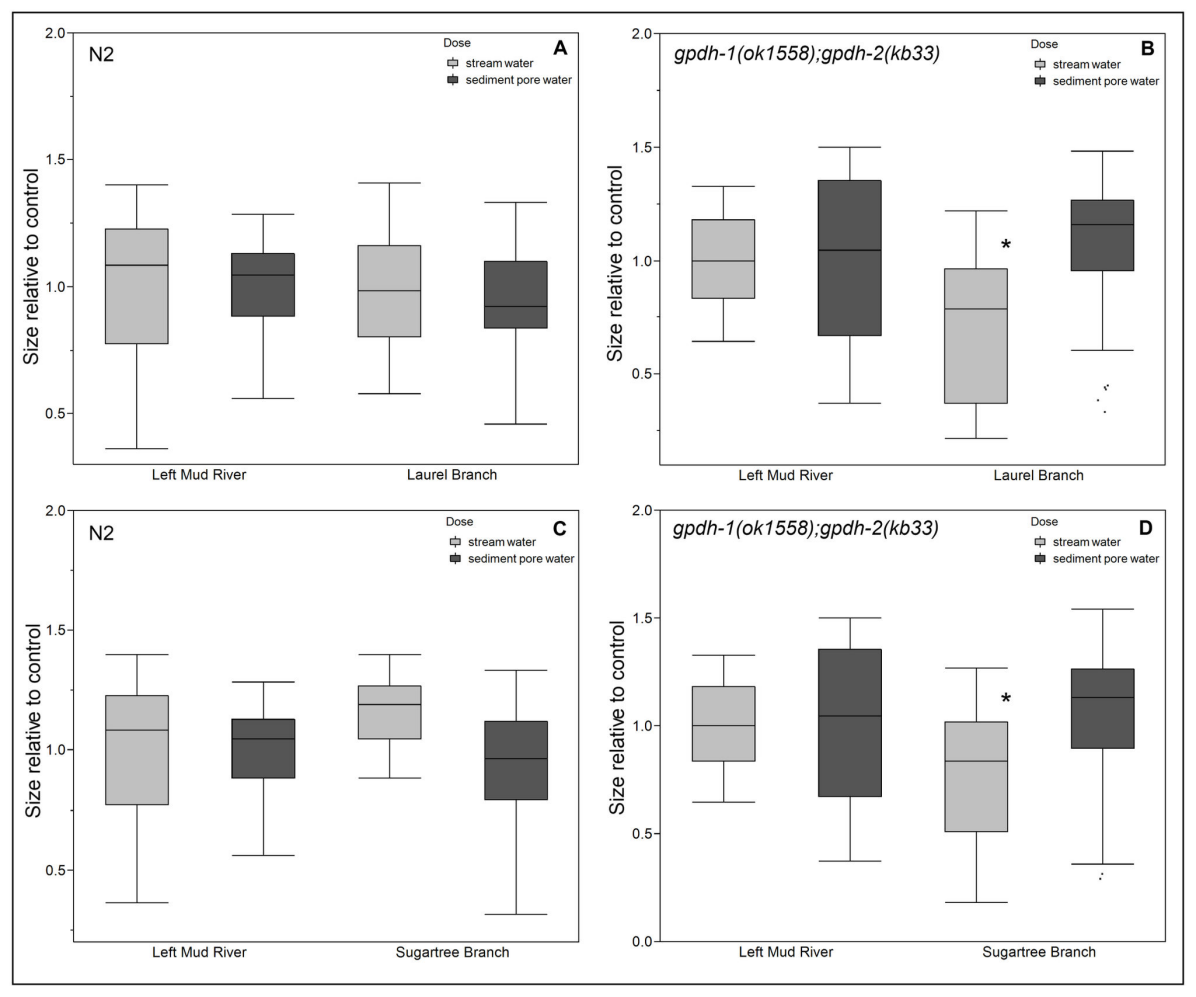
Toxicity of stream water is driven by osmotic stress. Stream water, but not sediment pore water, from the Mud River and its tributaries caused greater growth inhibition in osmotic stress-sensitive *gpdh-1*(*ok1558*);*gpdh-2*(kb33) deletion mutants in comparison to wild-type nematodes, demonstrated in samples collected from Sugartree Branch (**6A** and 6B p=0.0023n=23-55) and Laurel Branch (**6C** and 6D p=0.0004n=39-51), May 2012. Statistically significant difference from reference site indicated by asterisks. “Size” refers to the optical density (extinction) of each nematode and describes growth since the data shown is for size after three days of exposure. Each exposure was repeated at least two times separately.

Response to osmotic stress in nematodes is governed by two glycerol 3-phosphate dehydrogenases, *gpdh-1* and *gpdh-2*, which regulate synthesis and accumulation of glycerol in response to increased osmolarity. *gpdh-1* is strongly up-regulated in response to osmotic stress, whereas *gpdh-2* is constitutively expressed in several tissues and weakly up-regulated in response to osmotic stress. Mutant nematodes lacking either of these genes are slightly more sensitive to osmotic stress, and double knockouts are markedly more so, exhibiting growth inhibition in solutions with higher solute concentration [46]. We found that *gpdh-1*(*ok1558*);*gpdh-2*(*kb33*) mutants exhibited greater growth inhibition than wild-type nematodes following exposure to stream water, but not sediment pore water, from two sites with elevated conductivity(Figure 6) in comparison to reference site. Stream water from Mud River 6 and Mud River 7 taken in the same sampling trip did not cause strain-specific growth inhibition in keeping with the fact that the samples were taken shortly after flooding and had lower conductivities (growth data not shown, for conductivity data see Table S2).

Lethality was not observed for any of the exposure conditions.

Lastly, we used superoxide dismutase deficient nematodes to assess the potential role of oxidative stress in the toxicity of Mud River samples. *sod-3*(*gk257*) mutants, deficient in a mitochondria-localized iron/manganese superoxide dismutase and sensitive to oxidative stress [47,48], did not display greater growth inhibition than their wild-type counterparts when exposed to either water or sediment pore water from Mud River 6, 7, 9, 10 and 11 collected in June 2010 (data not shown).

## Discussion

The Mud River watershed is a complex and dynamic system. Growth data from wild-type nematodes exposed to samples taken from 3 different sampling trips (December 2010, April 2011, May 2012) reflects variability of water and sediment chemistry dependent on season, water level and influx of sulfates, metals, metalloids and assorted inorganic contaminants from the Hobet 21 mine. Although nematodes are more robust in response to many stressors than the native stream fauna that are typically first affected by mine runoff in mine-impacted ecosystems [49], wild-type nematodes were growth-inhibited upon exposure to both water and sediment pore water from the mining-impacted portions of the Mud River and its tributaries. As has been reported in previous research [50], impaired tributaries contributed to toxicity of water in the main stem of the river.

However, the major advantage of using *C. elegans* in these studies is the toolkit available for investigating mechanisms of toxicity. Nematodes employ a wide variety of stress-response mechanisms in order to maintain health in the face of adversity; reporter strains and knockout mutants allow the relationships between sensitivity to specific exposures and the mechanism of toxicity to be examined.

Using *mtl-2::GFP* expression as an endpoint, we detected no statistically significant increase in expression in comparison to either EPA water controls or nematodes treated with sediment pore water from our reference site. This might be explained by the absence of the types of metals that result in metallothionein induction. Metallothionein expression is induced most effectively by cadmium and other heavy metals, which are not typically present at elevated levels in mine-impacted Appalachian watersheds [11,14]. It is also possible that the lack of inductions reflect insufficient sensitivity of the reporter strain. However, *mtl-2*(*gk125*) worms grew comparably to wild-type in the presence of mining-impacted streamwater and sediment pore water. While *mtl-2*(*gk125*) deletion mutants are commonly employed as a metals-sensitive strain, recent research indicates that phytochelatin is more important for the mitigation of metal toxicity [22,42,51]. Our comparisons of the growth of *mtl-2*(*gk125*) and *pcs-1* (*tm1748*) deletion mutants support this data, since unlike the *mtl-2* (*gk125*) mutants, *pcs-1*(*tm1748*) mutants exhibited greater growth inhibition than wild-type nematodes when exposed to sediment from the Mud River (Figure S2).

While a genetic approach by itself cannot resolve which metals or metalloids are involved, our results do provide indirect evidence for an involvement of selenium. We found that *pcs-1*(*tm1748*) knockouts were more sensitive than wild-type to selenium, which is a known contaminant of the Mud River. While the relationship between phytochelatin and selenium remains unstudied in nematodes, algae [52] produce phytochelatin upon exposure to selenium, and selenium forms trisulfide linkages with phytochelatin *in vitro* [38]. The greater sensitivity of *pcs-1*(*tm1748*) knockout nematodes to sodium selenite in comparison to wild-type worms and a corresponding lack of induction of metallothionein expression after exposure to selenium, in concert with research by other groups demonstrating the greater importance of phytochelatin in comparison to metallothionein in the elimination of metals, suggest that phytochelatin is responsible for mitigating the effects of excess selenium in nematodes. A limitation of our experimental design is that most environmental selenium uptake is believed to occur via ingestion, rather than direct uptake from the medium. Although this is not something that we could test given the samples available to us, we note that the sediment pore water we used was not filtered and thus likely included ingestible particles. Future experiments using biofilms will be important to further our understanding of the potential role of ingestion of selenium.

Just as the impaired growth of *pcs-1*(*tm1748*) knockout nematodes in sediment pore water suggests the presence of biologically relevant levels of one or more metals or metalloids, the impaired growth of osmotic stress sensitive *gpdh-1*(*ok1558*);*gpdh-2*(*kb33*) knockouts implicates osmotic stress as another mechanism of toxicity. Previous research has demonstrated elevated levels of TDS in MTR/VF impacted Appalachian watersheds, including the Mud River [3,53]. Conductivity values exceeding the water quality benchmark of 300µs/cm^-1^ have been observed in the water of both the main stem and mining-affected tributaries (Table S2). Also relevant is the potential for mixture effects; mine output and the release of minerals via weathering are highly variable, and synergistic toxicity may be a factor [54-57].

Our interrogation via mutant studies of the differential growth inhibition between water and sediment pore water seen in wild-type nematodes reveals that the relevant mechanism of toxicity in the water column is primarily osmotic stress due to elevated TDS whereas metals or metalloids, most likely including selenium, are the most important toxicant seen in the sediment pore water. Positive growth effects caused by sediment pore water from the Left Fork are likely explained by sub-toxic quantities of selenium; while the Left Fork does not receive mine discharge, selenium in potentially biologically relevant quantities has been observed in biofilms collected from the Left Fork (Mariah Arnold, unpublished observation). Selenium in the mined portion of this system has been reported as typically ~1-20 µg/L in water [3]. However, ~90% of the selenium in the water column is present as selenate (Helen Hsu-Kim and Lusia Liu, personal communication) and is therefore unlikely to account for the growth inhibition that we observed, since selenate up to 150 µg/L had no effect on the growth of *C. elegans* in our experiments. On the other hand, sediment selenium was typically present as multiple species in this system, including selenite (Helen Hsu-Kim and Lusia Liu, personal communication), and might therefore contribute to growth inhibition as seen in our selenite growth assays (Figure 3).

We extend the previous successful use of *C. elegans* to investigate the environmental impact of metals mining [12] to the highly complex alkaline mine runoff associated with coal mining, illustrating the utility of *C. elegans* as a model organism for ecotoxicological research using environmental samples. Our work, in combination with chemical analyses and surveys of stream biota [3,6,58,59] supports the presence of biologically relevant levels of multiple toxicants in the mined portion of the Mud River system.

## Supporting Information

Figure S1 **Sediment pore water did not induce *mtl-2*::GFP.** Neither sediment pore water from Mud River and tributaries nor manganese and selenium controls caused a statistically significant increase in GFP expression in *mtl-2*::GFP transgenic nematodes in comparison to EPA water and Left Fork controls (n=18-147). Photos show uninduced *mtl-2*::GFP nematode (left) and cadmium-exposed *mtl-2*::GFP nematode (right).
(TIF)

Figure S2 **Sediment pore water caused greater growth inhibition in *pcs-1* knockout nematodes vs. *mtl-2* knockouts.** Sediment pore water from Mud River 6 (2A) and Mud River 7 (2B) caused greater growth inhibition in *pcs-1*(*tm1748*) deletion mutants (**2A** p=<0.0001 n=47-406, **2B** p=<0.0001 n=27-406) in comparison to wild-type nematodes, but not *mtl-2*(*gk125*) deletion mutants (**2A** p=0.3609 n=47-406, **2B** p=0.9487 n=27-406). Statistically significant difference from sediment-treated wild-type nematodes indicated by asterisks. “Size” refers to the optical density (extinction) of each nematode and describes growth since the data shown is for size after three days of exposure. Each exposure was repeated at least two times separately.
(TIF)

Table S1 **p values, Mann Whitney U test in comparison to EPA water controls.**
(DOCX)

Table S2 **Conductivity values, Mud River and tributaries.**
(DOCX)
