# Supplementary figures and images for: Assessing Different Mechanisms of Toxicity in Mountaintop Removal/Valley Fill Coal Mining-Affected Watershed Samples Using Caenorhabditis elegans
Source: PLoS One. 2013 Sep 16;8(9):e75329. doi: 10.1371/journal.pone.0075329 (PMC3774817; doi:10.1371/journal.pone.0075329)

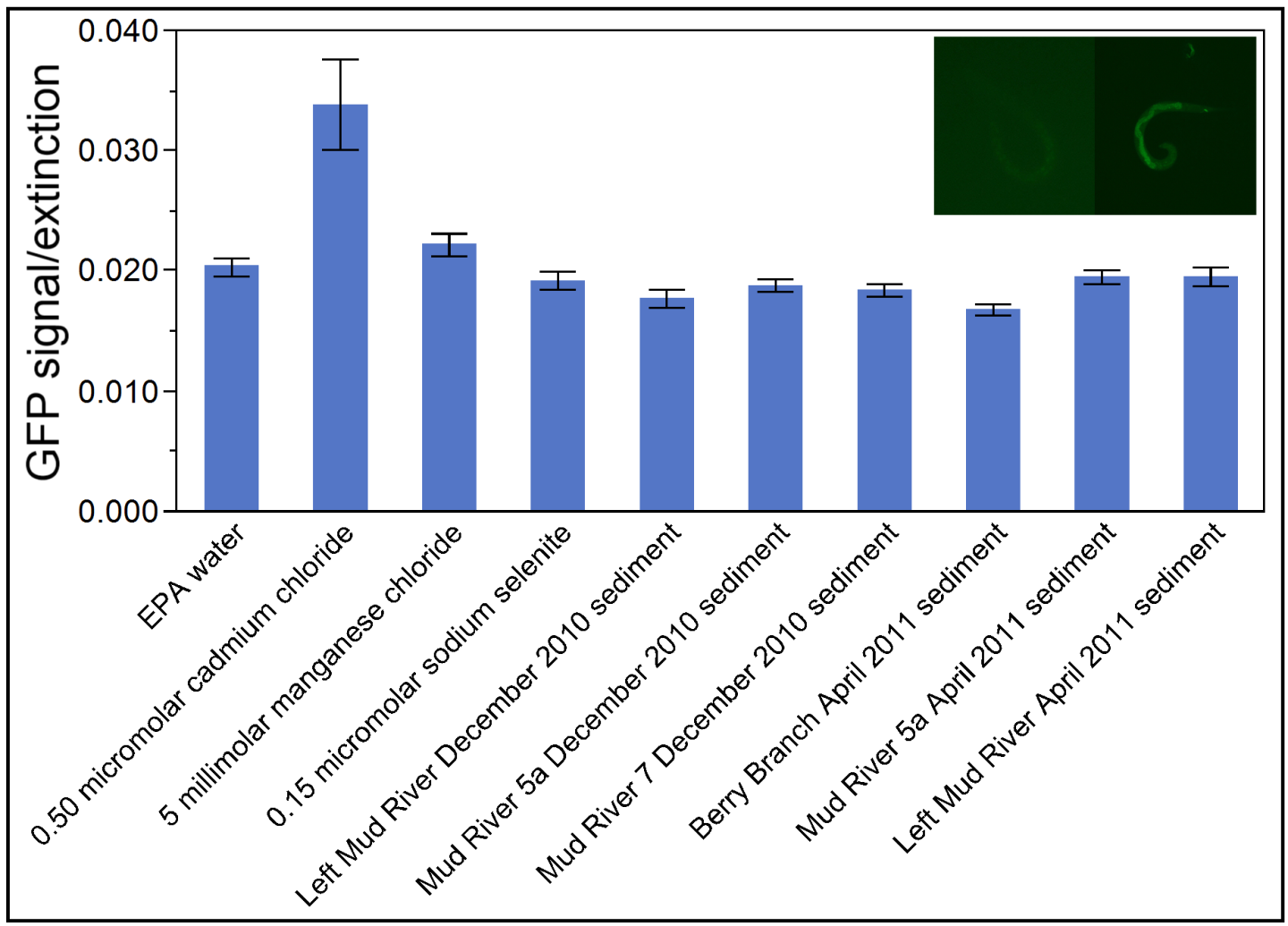

Supplement: Figure S1 — Sediment pore water did not induce mtl-2::GFP. Neither sediment pore water from Mud River and tributaries nor manganese and selenium controls caused a statistically significant increase in GFP expression in mtl-2::GFP transgenic nematodes in comparison to EPA water and Left Fork controls (n=18-147). Photos show uninduced mtl-2::GFP nematode (left) and cadmium-exposed mtl-2::GFP nematode (right). (TIF) [file pone.0075329.s001.tif]

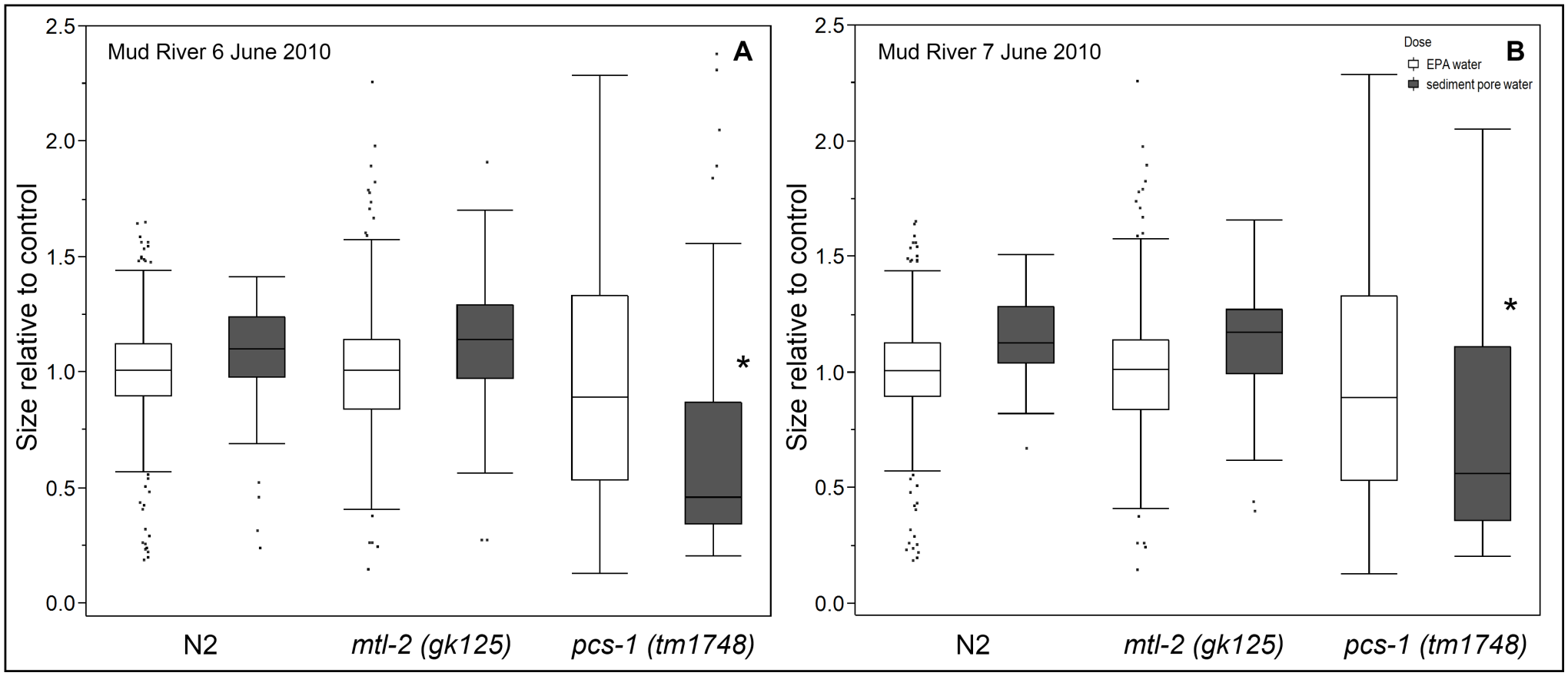

Supplement: Figure S2 — Sediment pore water caused greater growth inhibition in pcs-1 knockout nematodes vs. mtl-2 knockouts. Sediment pore water from Mud River 6 (2A) and Mud River 7 (2B) caused greater growth inhibition in pcs-1(tm1748) deletion mutants (2A p=<0.0001 n=47-406, 2B p=<0.0001 n=27-406) in comparison to wild-type nematodes, but not mtl-2(gk125) deletion mutants (2A p=0.3609 n=47-406, 2B p=0.9487 n=27-406). Statistically significant difference from sediment-treated wild-type nematodes indicated by asterisks. “Size” refers to the optical density (extinction) of each nematode and describes growth since the data shown is for size after three days of exposure. Each exposure was repeated at least two times separately. (TIF) [file pone.0075329.s002.tif]
