# Supplementary material for: Assessing Different Mechanisms of Toxicity in Mountaintop Removal/Valley Fill Coal Mining-Affected Watershed Samples Using Caenorhabditis elegans
Source: PLoS One. 2013 Sep 16;8(9):e75329. doi: 10.1371/journal.pone.0075329 (PMC3774817; doi:10.1371/journal.pone.0075329)
Supplement: Table S1 — p values, Mann Whitney U test in comparison to EPA water controls. (DOCX) [file pone.0075329.s003.docx]

**Table S1. *p* values, Mann Whitney U test in comparison to EPA water controls.**

| **April 2011** |  |  |  |
| --- | --- | --- | --- |
| Site | Water | Sediment Pore Water | Water vs. Sediment Pore Water |
|  |  |  |  |
| **Left Mud River (reference)** | 0.5451 | 0.0327 | 0.1775 |
| **Mud River 5** | 0.0008 | 0.1339 | 0.3939 |
| **Stanley Fork** | <0.0001 | 0.3914 | <0.0001 |
| **Mud River 5a** | <0.0001 | 0.0031 | 0.0237 |
| **Mud River 6** | <0.0001 | 0.0001 | <0.0001 |
| **Laurel Branch** | <0.0001 | 0.0069 | 0.8584 |
| **Mud River 7** | 0.0029 | 0.005 | 0.7772 |
| **Mud River 8b** | <0.0001 | 0.0023 | 0.0741 |
| **Connelly Branch** | <0.0001 | ---- | ---- |
| **Mud River 8** | <0.0001 | 0.0902 | 0.0042 |
| **Mud River 10a** | <0.0001 | 0.3675 | <0.0001 |
| **Mud River 10** | <0.0001 | 0.3877 | 0.0001 |
| **Berry Branch** | <0.0001 | <0.0001 | 0.7691 |
|  |  |  |  |
| p-value cutoff for statistical significance | 0.0038 | 0.0042 | 0.0042 |

Comparison of optical density (extinction) of wild-type nematodes treated with steam water and sediment pore water to EPA water-treated controls. The Bonferroni-corrected p-value cut-off for significance based on 13 pairwise comparisons is p < o.oo38.
