# Supplementary material for: Assessing Different Mechanisms of Toxicity in Mountaintop Removal/Valley Fill Coal Mining-Affected Watershed Samples Using Caenorhabditis elegans
Source: PLoS One. 2013 Sep 16;8(9):e75329. doi: 10.1371/journal.pone.0075329 (PMC3774817; doi:10.1371/journal.pone.0075329)
Supplement: Table S2 — Conductivity values, Mud River and tributaries. (DOCX) [file pone.0075329.s004.docx]

**Table S2. Conductivity values, Mud River and tributaries.**

|  | | | | | | | | | | | |
| --- | --- | --- | --- | --- | --- | --- | --- | --- | --- | --- | --- |
|  | **December 8-10 2010** | **April 12-15 2011** | **May 1 2012** |  |  |  |  |  |  |  |  |
|  | conductivity, µs | conductivity, µs | conductivity, µs |  |  |  |  |  |  |  |  |
|  |  |  |  |  |  |  |  |  |  |  |  |
| **Left Fork** |  | 179 | 55 |  |  |  |  |  |  |  |  |
| **Mud River 5** | 1057 |  |  |  |  |  |  |  |  |  |  |
| **Stanley Fork** | 1814 |  |  |  |  |  |  |  |  |  |  |
| **Mud River 5a** | 1315 |  |  |  |  |  |  |  |  |  |  |
| **Sugartree Branch** | 1412 |  | 868 |  |  |  |  |  |  |  |  |
| **Mud River 6** | 1326 |  | 366 |  |  |  |  |  |  |  |  |
| **Laurel Branch** | 1411 |  | 778 |  |  |  |  |  |  |  |  |
| **Mud River 7** | 1324 | 850-1500 | 380 |  |  |  |  |  |  |  |  |
| **Mud River 8b** | 1350 | 698 |  |  |  |  |  |  |  |  |  |
| **Connolly Branch** | 2120 | 1889 |  |  |  |  |  |  |  |  |  |
| **Mud River 8** | 1586 | 899 |  |  |  |  |  |  |  |  |  |
| **Mud River 10a** | 1592 | 837 |  |  |  |  |  |  |  |  |  |
| **Mud River 10** | 1660 | 826 |  |  |  |  |  |  |  |  |  |
| **Berry Branch** | 1105 | 760 |  |  |  |  |  |  |  |  |  |

Conductivity measurements taken in the field. US EPA water quality guidelines name 300 µs /cm^2^ as the cutoff for impairment.
